# Astragaloside III Enhances Anti-Tumor Response of NK Cells by Elevating NKG2D and IFN-γ

**DOI:** 10.3389/fphar.2019.00898

**Published:** 2019-08-13

**Authors:** Xingmeng Chen, Xi Chen, Junxiao Gao, Han Yang, Yue Duan, Yuxin Feng, Xin He, Xiaoqun Gong, Hanjie Wang, Xiaoli Wu, Jin Chang

**Affiliations:** ^1^Tianjin Engineering Center of Micro Nano Biomaterials and Detection Treatment Technology, Collaborative Innovation Center of Chemical Science and Engineering, School of Life Sciences, Tianjin University, Tianjin, China; ^2^College of Pharmaceutical Engineering of Traditional Chinese Medicine, Tianjin University of Traditional Chinese Medicine, Tianjin, China; ^3^Tianjin Key Laboratory of Modern Chinese Medicine, Tianjin University of Traditional Chinese Medicine, Tianjin, China; ^4^School of Traditional Chinese Medicine, Guangdong Pharmaceutical University, Guangzhou, China

**Keywords:** Astragaloside III, natural killer cells, anti-tumor, natural killer group 2D, IFN-γ

## Abstract

Natural killer (NK) cells play an irreplaceable role in the development of colon cancer, in which antitumor function of NK cells was impaired. Astragaloside III is a natural compound from Astragalus that has been shown to have immunomodulatory effects in various systems. However, few studies have evaluated the antitumor effects of Astragaloside III through stimulating systemic immunity and regulating NK cells. In this study, flow cytometry, immunohistochemical analysis, and immunofunctional assays were performed to elucidate the functions of Astragaloside III in restoring antitumor function of NK cells. We demonstrated that Astragaloside III significantly elevated the expression of natural killer group 2D (NKG2D), Fas, and interferon-γ (IFN-γ) production in NK cells, leading to increased tumor-killing ability. Experiments in cell co-culture assays and CT26-bearing mice model further confirmed that Astragaloside III could effectively impede tumor growth by increasing infiltration of NK cells into tumor and upregulating the antitumor response of NK cells. We further revealed that Astragaloside III increased IFN-γ secretion of NK cells by enhancing the expression of transcription factor T-bet. In conclusion, the effective anti-tumor function of Astragaloside III was achieved through up-regulation of the immune response of NK cells and elevation of NKG2D, Fas, and IFN-γ production.

## Introduction

Colorectal cancer (CRC) is the second leading cause of cancer-related death and led to 0.55 million deaths in 2018 (Ferlay et al., 2015; Bray et al., 2018). Although several conventional therapies are used for the treatment of CRC, the mortality rates of patients rank from 13% to 89% (Siegel et al., 2017), indicating the limitations of current treatments and urgent needs for more effective therapies. Recently, remarkable progress was made in immunotherapy for the treatment of CRC (Brahmer et al., 2012; Topalian et al., 2012; Eggermont et al., 2016). Among them, activating the function of innate immune cells-natural killer (NK) cells has been shown to exhibit strong anti-tumor effects (Hsu et al., 2018).

NK cells are bone marrow-derived innate immune cells that play critical roles in the first line of defense against cancer (Pahl and Cerwenka, 2017). NK cells express distinct markers in different types of mice, for instance, CD49b in BALB/c and NK1.1 in B16 mice. Despite this, cytotoxity function of NK cells is tightly regulated by the same family of activating (e.g., NKG2D and Fas) and inhibitory receptors (Backes et al., 2018). The lysis of cancer cells is triggered by low expression of ligands for NK cell inhibitory receptors, such as NKG2A, killer cell Ig-like receptors, and CD244 (Lanier, 2015; Muntasell et al., 2017), in combination with high expression of NK cell-activating receptors, such as NKG2D, NKp30, NKp46, and natural cytotoxicity receptors (Pahl and Cerwenka, 2017). The central roles of NKG2D, a highly conserved receptor that mediates NK cell-based immunotherapy, are further demonstrated by various studies showing that tumor cells adopt multiple approaches to evade NKG2D-mediated surveillance (Zhu et al., 2018). In a variety of colorectal cancer cells and primary colon carcinoma, NKG2D can specifically recognize NKG2D ligands, namely, MICA, MICB, and ULBP, which are frequently expressed on colon tumor cells but with restricted expression in normal tissues (Nausch and Cerwenka, 2008). Clinical investigations have shown that high levels of NKG2D ligands on tumor cells predict favorable prognosis (Lanier, 2015), whereas reduced expression of NKG2D usually correlates with strong prognostic relevance in patients (McGilvray et al., 2009). Thus, enhancing the response of NK cells will be a new and promising immunotherapeutic strategy for treatment of colon tumor. However, in patients with colon cancer, cytotoxic activity of NK cells is eluded by decreasing the expression of NKG2DL, increasing immunosuppressive cytokines, and overexpression of NK cell inhibitory receptors (Tallerico et al., 2013). The immunosuppressive tumor microenvironment significantly down-regulates NKG2D expression and IFN-γ production of NK cells (Vitale et al., 2014). Thus, to restore the impaired function of NK cells, both the expression of stimulatory receptors (e.g., NKG2D) and the cytokine production of NK cells need to be increased in tumors.

NK cells can indirectly influence anticancer or antimetastatic activities by secreting various effector molecules, such as IFN-γ, Granzyme B, and perforin (Zundler and Neurath, 2015). As previously reported, NK cells are the cardinal IFN-γ producers that are conducive to the direct elimination of tumor and lead to the activation of other immune cells, such as T and B cells (Xu et al., 2017). IFN-γ produced by NK cells can arrest tumor cell proliferation, tumor angiogenesis, and multistage carcinogenesis (Konjevic et al., 2016). Meanwhile, it also can induce the expression of ligands for NK cell receptors on cancer cells and further enhance tumor immunosurveillance (Morvan and Lanier, 2016). In view of skewed production of IFN-γ by NK cells in tumor microenvironment, it is urgent to seek drugs that can restore the IFN-γ secretion of NK cells. As we know, the expression of IFN-γ is regulated by the transcription factor T-bet (Spits et al., 2016). Once activated by cytokines like IL-2, IL-12, and IL-15, increased expression of T-bet can significantly induce large amounts of IFN-γ secreted by NK cells (Zhang et al., 2011). Thus, by using other compound to activate antitumor response of NK cells will be a promising way for the treatment of colon cancer.

Astragalus is a well-known traditional Chinese herb, and it is based on an herbal mixture commonly used together with chemotherapy for treatment of cancer (Auyeung et al., 2016). Accumulating studies have shown that Astragalus displays great potential in immunomodulatory properties and antitumor activities (Wang et al., 2002; McCulloch et al., 2006; Wan et al., 2013). According to the structure of compound, Astragalus has been divided into several major components. Most of the studies focus on the efficacy of Astragaloside components in regulating the adaptive immune response for tumor treatment. Astragalosides I and II significantly increased IL-2 and IFN-γ secretion by CD4^+^ T cells (Lee et al., 2017). Astragaloside II can specifically trigger T-cell activation and reduce multidrug resistance for hepatic cancer chemotherapy (Wan et al., 2013). Astragaloside IV is reported to inhibit tumor progression by downregulating the percentage of regulatory T (Treg) cells and upregulating the percentage of cytotoxic T lymphocytes (Li et al., 2017). It also significantly inhibited the migration and invasion of tumor by reducing the production of cytokine IL-6, TNF-α, and TGF-β (Li et al., 2017). Meanwhile, Astragaloside VII could increase the production of Th1 cytokines (IL-2 and IFN-γ) (Nalbantsoy et al., 2011). However, few studies focus on the effect of Astragaloside III in tumor treatment, especially their regulatory ability in activating innate immune response (eg, NK cells).

In this study, we analyzed and characterized the regulatory function of Astragaloside III on activating anti-tumor response of NK cells and showed that Astragaloside III could significantly enhance anti-colon cancer capability of NK cell through increasing the expression of NKG2D and IFN-γ in NK cells both *in vivo* and *in vitro*. Furthermore, increased IFN-γ was regulated by the elevation of T-bet in transcription levels.

## Materials and Methods

### Mice

All experiments were performed with female mice (6–8 weeks). BALB/c mice were purchased from the Beijing HFK Bioscience company (Beijing, China) and maintained in the Tianjin International Joint Academy of Biomedicine under specific pathogen-free conditions. All protocols conformed to the Animal Ethics Committee of the Tianjin International Joint Academy of Biotechnology and Medicine.

### Reagents

Astragaloside III was purchased from Dalian Meilun Biological Technology Co., Ltd. (Dalian, China). All flow cytometry antibodies were purchased from BioLegend (BioLegend, USA). RPMI-1640 medium, trypsin, penicillin-streptomycin, and fetal bovine serum were purchased from Gibco (Gibco USA). Recombinant mouse IL-2 and IL-12 were purchased from R&D Systems (Minneapolis, MN, USA). CFSE Cell Proliferation Kit was purchased from ThermoFisher (ThermoFisher, USA). Red blood cell lysis buffer and 4% paraformaldehyde DAPI, DMSO were all purchased from Solarbio (Beijing, China), Quantscript RT Kit, SYBR Premix Hotmaster Taq were from Tiangen (Beijing, China). All primary antibodies and fluorophores were purchased from Zhongshan Golden Bridge Bio-technology Co., Ltd. (Beijing, China).

### NK Cells Stimulation and Coculture Experiments

Spleenocytes from BALB/c mice were cultured in a 96-well plate at density of 4 × 10^6^ cells/ml; IL-2 (2 ng/ml) and IL-12 (20 ng/ml) were used to differentiate the NK cells profile. Astragaloside III was added at different concentration ranges from 4 nmol/L (nM) to 40 nM. After 48 h, cells were harvested for further detection.

For tumor cell coculture assay, CT26 was labeled with certified functional safety expert (CFSE) as previously reported (Quah and Parish, 2012). Then Astragaloside III stimulated (20 nM) spleenocytes or isolated NK cells were cocultured with CFSE labeled tumor cells (E:T = 1:1) into 96-well plates. After 6 h, proliferation ratio of CT26 as well as NKG2D expression and IFN-γ production of NK cells were checked.

### Intracellular Staining

To detect cytokine production of NK cells, spleenocytes were harvested 24 h after Astragaloside III stimulation. For intracellular IFN-γ staining, cells were placed in a 48-well plate and incubated with PMA (300 ng/ml; Sigma, St. Louis, MO) and ionomycin (1 μg/ml; Sigma-Aldrich) for 6 h; monensin was added 2 h before cells harvested. Then cells were washed with phosphate-buffered saline (PBS), fixed, permeabilized, and stained with IFN-γ as described previously (Andersen et al., 2017). Those cells were examined and analyzed with FACS Calibur and FlowJo software (Tristar, San Carlos, CA).

### Tumor Mouse Model and Treatment

To establish tumor model, 5 × 10^5^ CT26 cells were subcutaneously injected into the right armpit of BALB/c mice. Treatments were initiated when tumors reached a mean group size of approximately 100 mm^3^. Control group was injected with PBS, and treatment group was intravenously injected with 50 mg/kg Astragaloside III mixed with 10% PEG-200 and 10% ethanol. Astragaloside III was given 2-day intervals five times. The tumor volume was measured every other day by a caliper and calculated as V = W^2^L/2, where W and L represent the shortest and longest diameters separately. Three weeks later, mice from two groups were sacrificed. Tissues were collected for further functional studies.

### Histology

Tumor, heart, liver, spleen, lung, and kidney tissues were harvested, fixed in 10% formalin, and embedded in paraffin. Five-micrometer sections were stained with hematoxylin and eosin, and the images were acquired on a Leica DM3000 microscope. The sections of tissues were quantified by the total area using Image-Pro Plus 6.0 software (Media Cybemetics, Silver Spring, MD, USA).

### Systemic Immune Assessment on Flow Cytometry

Tumor tissue, lymph node, and spleen were collected from two groups, grinded into suspension and stained with antibodies of NKG2D, CD8, CD3, and CD49b. After staining for 15 min. Cells were washed with PBS, fixed, and analyzed using FACS Calibur and FlowJo software. For IFN-γ staining, steps are the same as above.

### Immunofluorescence Staining

After the process of dewaxing, antigen regain, and permeabilization, tumor tissue sections were blocked in 5% Bovine serum albumin (BSA) at room temperature for 15 min. The tumor sections were treated with mouse anti-CD3 primary antibody (dilution 1:200), Alex 488-conjugated goat anti-mouse secondary antibody (dilution 1:200) for CD3, rabbit anti-CD49b primary antibody (dilution 1:100), and Rhodamine-conjugated goat anti-rabbit secondary antibody (dilution 1:200) for CD49b; sections were incubated at 4°C overnight. After 24 h, cell nuclei were stained with DAPI. Images were captured using laser-scanning confocal microscope (Zeiss Germany).

### Real-Time PCR (RT-PCR) for Gene Transcription

Total RNA was extracted from tumor tissues and reverse-transcribed. mRNA expression was quantified using SYBR Premix Hotmaster Taq (Tiangen, Beijing, China), and glyceraldehyde-3-phosphate dehydrogenase (GADPH) gene expression was used as an internal control. The primer sequences used were as follows:

IFN-γ
  forward: 5′-ATG AAC GCT ACA CAC TGC ATC-3′,
  reverse: 5′-CCA TCC TTT TGC CAG TTC CTC 3′;
IL-12
  forward: 5′-TCT CCC ACA GGA GGT TTC TG 3′;
  reverse: 5′-ACA GAG TTC CAG GCC ATC AA-3′,
TNF-α
  forward: 5′-CTA CTG AAC TTC GGG GTG AT-3′,
  reverse: 5′- CAG GCT TGT CAC TCG AAT T-3′;
T-bet
  forward: 5′-TTT CAT TTGGGA AGC TAA AG-3′,
  reverse: 5′-GGC TGG TAC TTG TGG AGA GA-3′;
IL-6
  forward: 5′-CTG CAA GAG ACT TCC ATC CAG-3′,
  reverse: 5′-AGT GGT ATA GAC AGG TCT-3′;
GATA-3
  forward: 5′-GCT TCA CAA TAT TAA CAG ACC C-3′,
  reverse: 5′-TTA AAC GAG CTG TTC TTG GG-3′;
GAPDH
  forward: 5′-CGG AGT CAA CGG ATT TGG TCG TAT-3′,
  reverse, 5′-AGC CTT CTC CAT GGT GGT GAA GAC-3′.

### Statistics

All data are presented as the mean ± SD. Statistical significance was evaluated by a two-tailed unpaired Student’s *t*-test using InStat version 6.0 software for Mac (GraphPad, San Diego, CA, USA). Throughout the text, figures, and legends, the following terminology is used to show statistical significance: *P < 0.05; **P < 0.01, and ***P < 0.001.

## Results

### Astragaloside III Elevated the Expression of NKG2D and IFN-γ in NK Cells *in Vitro*

**Figure 1:**
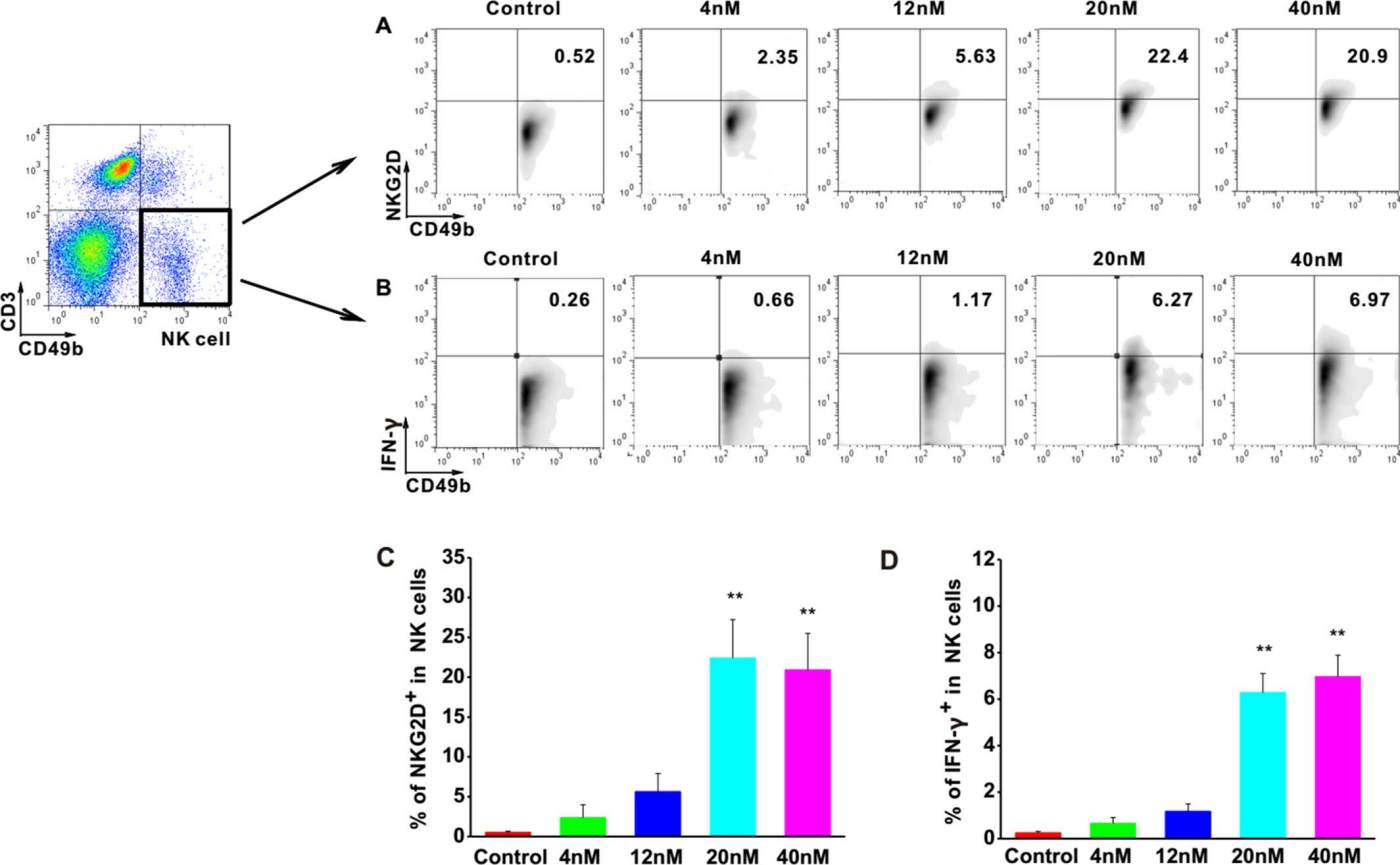
Astragaloside III significantly elevate expression of NKG2D and IFN-γ in NK cells *in vitro*. A, B, Representative dot plots show the expression of NKG2D **(A)** and IFN- γ **(B)** on NK cells after stimulated with different concentrations of Astragaloside III (gated on CD49^+^ CD3^-^ cells). C, D, Statistical analysis of the frequencies of IFN- γ **(C)** and NKG2D **(D)** expression on NK cells in control and different dose of Astragaloside III stimulation groups (0, 4, 12, 20, 40 nM). All data are presented as the means ± standard errors. ** *P* < 0.01.

To investigate the effects of Astragaloside III on NK cells, the expressions of NKG2D and IFN-γ were examined in NK cells upon stimulation with Astragaloside III. The cell population of CD49b^+^ CD3^-^ was determined as NK cells. FACS analysis revealed increased levels of NKG2D and IFN-γ in these cells upon treatment with different concentrations of Astragaloside III in comparison with control groups (**Figures 1A, B**). Statistical analysis confirmed that the increase of NKG2D and IFN-γ was significant in 20 and 40 nM stimulation groups, compared with control, and 4 and 12 nmol stimulation group (**Figures 1C, D**). Furthermore, the secretion of IFN-γ by NK cells isolated from spleen significantly increased in the treatment group than that in the control group (**Figure S1**), which indicated the direct activation of Astragaloside III on NK cells. These data suggested that Astragaloside III can activate the function of NK cells by elevating the expression of NKG2D and IFN-γ *in vitro*.

### Astragaloside III Induced CT26 Cell Death by Activating NK Cells

**Figure 2:**
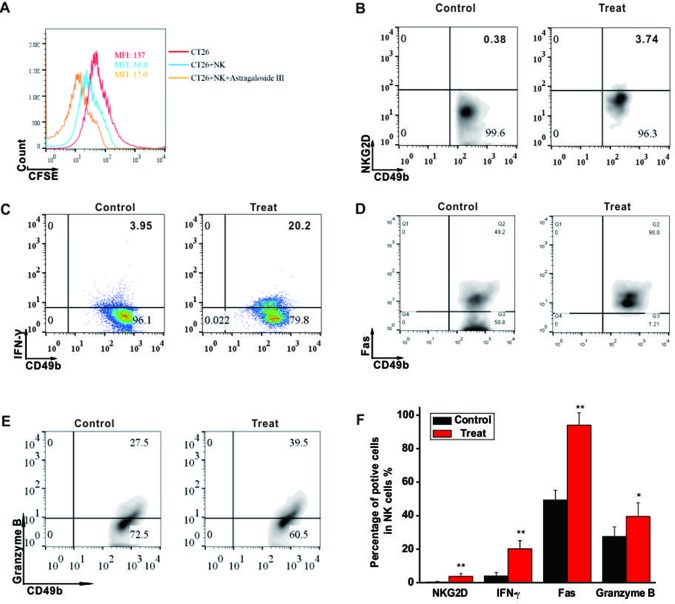
Astragaloside III induced CT26 cell death through activating NK cell response. **(A)**, Flow cytometry results represent the mean fluoresce intensity (MFI) of CFSE-labeled CT26 (red line), CT26 unstimulated NK cells (blue line) and CT26+NK cells +Astragaloside III (yellow line). **(B, C)**, Percentage of NKG2D^+^ NK cells **(B)** and IFN-γ^+^ NK cells **(C)** in coculture system with (treat) or without (control) Astragaloside III. D, E, The expressions of Fas **(D)** and Granzyme **(E)** on NK cells after co-cultured with CT26 cells were determined by flow cytometry. **(F)** Statistic data regarding the expression of NKG2D, IFN-γ, Fas and Granzyme on NK cells in control and treatment groups. Data are presented as means ± standard errors. * *P* < 0.05, ** *P* < 0.01.

To investigate the cytotoxic function of NK cells to CRC tumors, CT26 cell co-culture system was established. As shown in **Figure 2A**, mean fluoresce intensity (MFI) of CFSE-labeled CT26 (red line, 137) was decreased when the cells were co-cultured with unstimulated NK cells (blue line, 38.4), and more drastically decreased with NK cells stimulated by Astragaloside III (yellow line, 17). However, Astragaloside III had no cytotoxity effect on CT26 (**Figure S2**), so antitumor effect of Astragaloside III was through the activation of NK cells *in vivo*. As Astragaloside III-activated NK cells media could significantly increase the expression of AnnexinV (apoptosis marker) and 7-AAD (death marker) on CT26 cells when compared with control (**Figure S4A**), the effect of Astragaloside III on anti-tumor mechanism of NK cells was further detected. Consistent with prior experiments, expression of NKG2D and IFN-γ on NK cells also increased in Astragaloside III group than that in the control group (**Figures 2B, C**). Meanwhile, Astragaloside III significantly increased the expressions of Fas and Granzyme B (**Figures 2D, E**) but not perforin (**Figure S3A**) on NK cells. Statistical analysis was used to further prove that observation (**Figure 2F**). However, the expression of NKG2A, an inhibition receptor of NK cells, had no significance between the control and Astragaloside III group (**Figure S4B**). These results suggested that Astragaloside III can effectively inhibit the proliferation of CT26 cells through elevation of NKG2D, Fas, Granzyme B, and IFN-γ expression, and activating NK cells.

### Astragaloside III Significantly Inhibited the Growth of CT26 in Mouse Model

**Figure 3:**
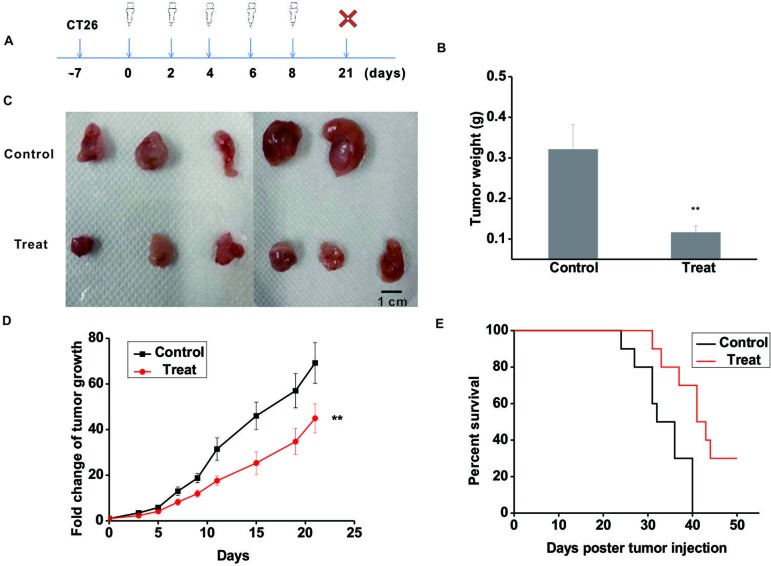
Astragaloside III significantly inhibits the growth of CT26 in tumor-bearing mice model. **(A)** Experimental schematics of CT26-bearing mice model. Astragaloside III was injected (*i.v.* 50 mg/kg) in different time points for treatment of CT26-bearing mice. **(B)** Tumor weights of control and Astragaloside III treatment group. **(C)** Physical photographs of tumors in control and Astragaloside III treatment groups. The scale is 1 cm. **(D)** Tumor growth curves of control (n = 5) and Astragaloside III treatment group (n = 6). **(E)** Survival rate of control and Astragaloside III treatment group. Data are presented as the means ± standard errors. ** *P* < 0.01.

To further validate the anti-tumor function of Astragaloside III, CT26 tumor model was established. The experimental scheme is shown in **Figure 3A**. Seven days post-CT26 plantation, Astragaloside III was injected (*i.v.*) every 2 days for five times. After 3 weeks, mice were sacrificed. As shown in **Figure 3B**, the tumor weight of the treatment group was significantly lower than that of the control group (**Figure 3B**, *P* < 0.01). Meanwhile, tumor sizes of the Astragaloside III treatment group were smaller than that of the control group (**Figure 3C**, scale: 1 cm). Fold changes of tumor volume were further examined. As shown in **Figure 3D**, fold changes of tumor volume increase were significantly lower in the Astragaloside III treatment groups than that of the control group (*P* < 0.01). In addition, Astragaloside III could significantly prolong the survival rate of CT26-bearing mice than those of the control group (**Figure 3E**). In the treatment group, there were still 30% of mice that survived by day 50. All mice in the control group, however, were found dead within 40 days. In summary, Astragaloside III effectively inhibited the growth of CT26 in mouse model.

### Astragaloside III Effectively Recruited and Activated NK Cells in Tumor Site

**Figure 4:**
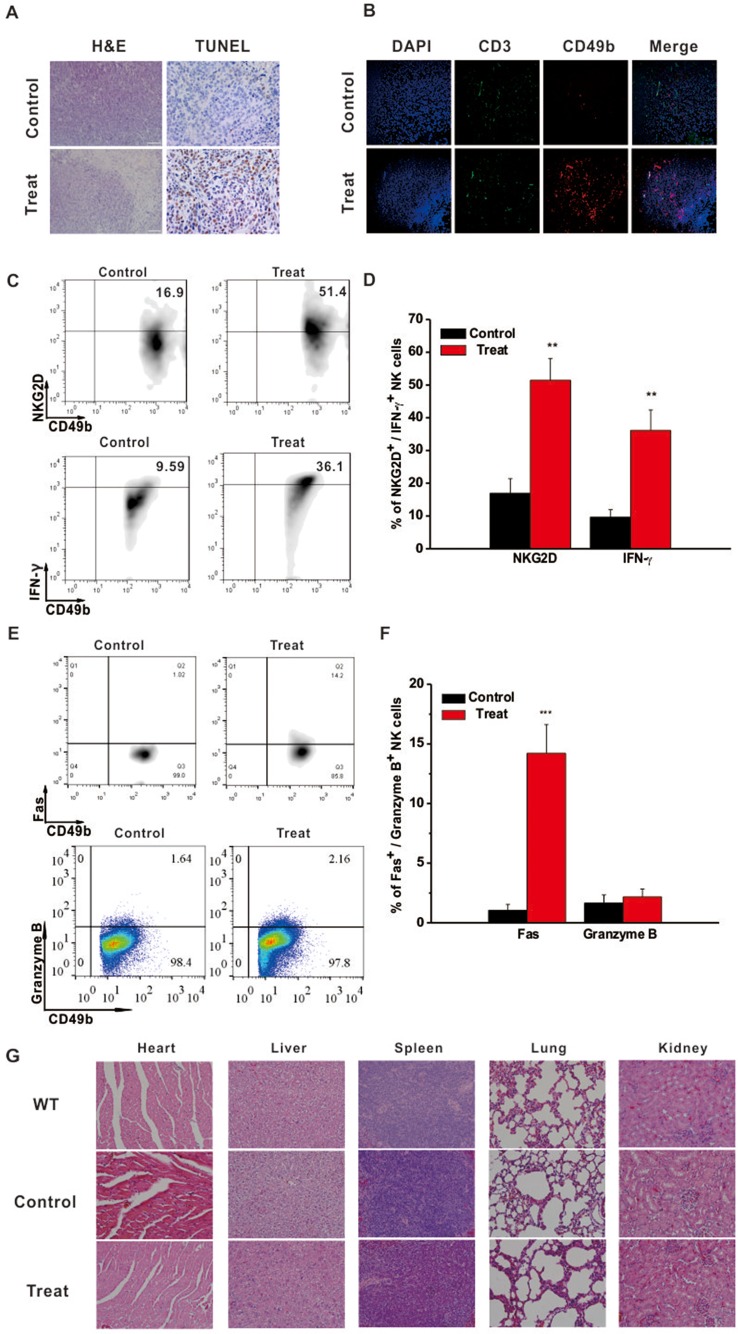
Astragaloside III recruited and activated NK cells in tumor site without side effect. **(A)** Tumor H&E (200×) and TUNEL (400×) stained images of control and Astragaloside III treatment group. **(B)** Immunofluorescence image showed the expression of CD49 (red) and CD3 (green) in tumor of control and Astragaloside III treatment group. Arrowheads in control group indicated NK cells (CD49^+^ CD3^-^, red only). **(C, D)** Representative dot plots **(C)** and statistic data **(D)** depict the expression of NKG2D and IFN-γ on NK cells in tumor of two groups. Data were presented as means ± standard errors. ** *P* < 0.01. **(E, F)** Expressions of Fas and Granzyme on NK cells from CT26 tumor model were determined by flow cytometry. **(F)** Statistic data regarding the expression of Fas and Granzyme on NK cells in control and treatment groups. *** *P* < 0.001. **(G)** H&E-stained images of different organs in wild type, control, and Astragaloside III treatment groups (200×).

Intratumoral immune status was examined to further elucidate the underlying anti-tumor mechanism of Astragaloside III. Hematoxylin and eosin (H&E) staining showed that tumors from the Astragaloside III-treated group exhibited obvious structural destruction and necrosis than those from the control group. TUNEL staining was further performed in tumors of the two groups. Significantly more TUNEL-positive cells were found in tumors of the treatment group than the control group (**Figure 4A**). To further detect the changes of infiltration cells in tumors, immunofluorescence experiments were performed. As shown in **Figure 4B**, more NK cells (CD49^+^ CD3^-^, red only) were infiltrated into the tumors of the Astragaloside III treatment group (red, CD49; green, CD3; blue, DAPI indicated the nucleus) than the control group. We did not observe such changes with CD3^+^ T cells (green). Moreover, Astragaloside III dramatically enhanced the expression of NKG2D and IFN-γ on NK cells in tumors (**Figure 4C**). Statistical analysis further confirmed this observation (**Figure 4D**). Meantime, expression of Fas (**Figure 4E**) but not Granzyme B (**Figure 4E**) and Perforin (**Figure S3B**) was significantly increased by NK cells when treated with Astragaloside III. Statistical data support this result (**Figure 4F**). H&E staining was also performed with major organs (heart, liver, spleen, lung, and kidney), revealing that Astragaloside III induced no obvious toxic side effects in the CT26-bearing mice (**Figure 4G**). These results suggested that Astragaloside III could enhance the anti-tumor effects of NK cells in colon cancer without obvious toxicity.

### Astragaloside III Activated the Function of NK Cells in Different Immune Organs

**Figure 5:**
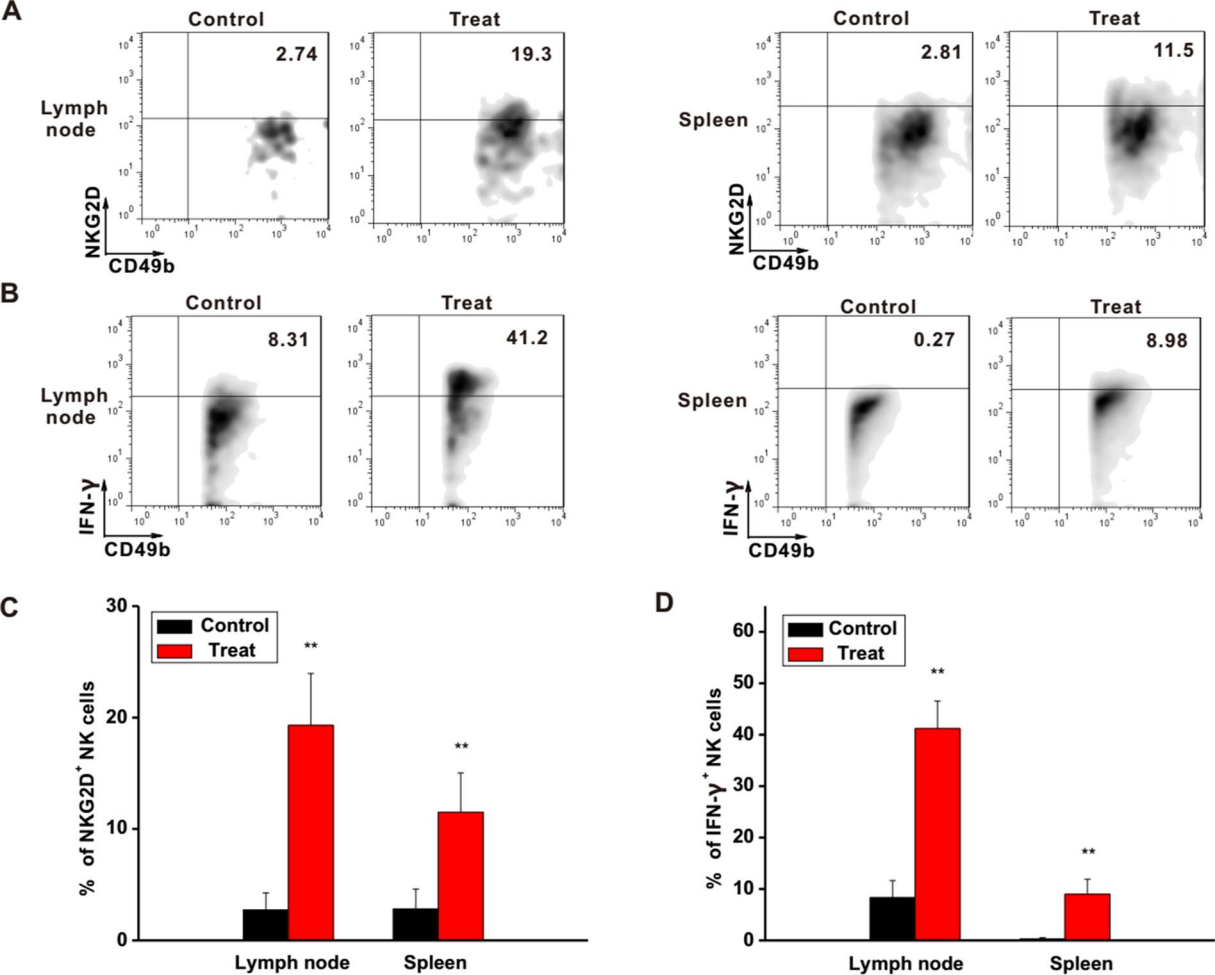
Astragaloside III active NK cells in different immune organs. **(A, B)**, Representative dot plots showed the expression of NKG2D **(A)** and IFN-γ **(B)** on NK cells in tumor lymphocyte and spleen. **(C, D)**, Statistical analysis of the frequencies of NKG2D **(C)** and IFN-γ **(D)** expression on NK cells in control and Astragaloside III-treated groups. All data are presented as the means ± standard errors. ** *P* < 0.01.

To further investigate the regulatory functions of Astragaloside III in the immune system, the status of NK cells in lymph node and spleen was examined. In the lymph nodes and spleens of Astragaloside-treated mice, expression levels of NKG2D and IFN-γ on NK cells were clearly higher than that of the control group (**Figures 5A, B**). Statistical analysis further confirmed this observation (**Figures 5C, D**, *P* < 0.01). In addition, IFN-γ level in serum was also increased after treatment with Astragaloside III (**Figure S5**). Based on these, Astragaloside III appeared to be able to alter the function of NK cell in systemic immune organs.

### Astragaloside III Increased Cytokine Production and T-Bet Expression in Tumor

**Figure 6:**
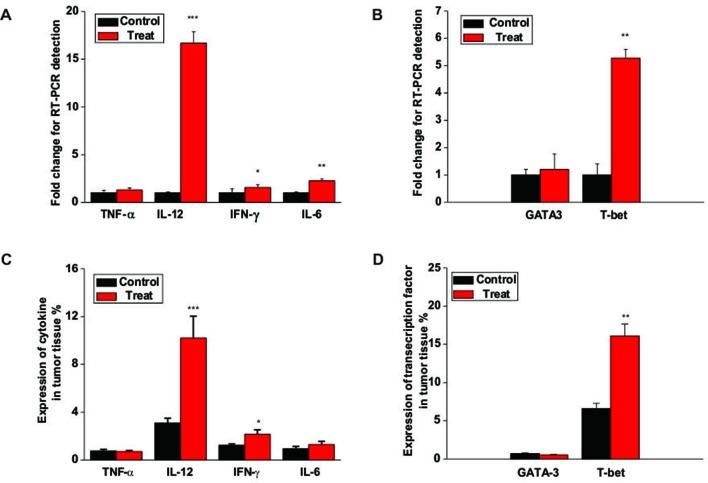
Astragaloside III enhances cytokine production and T-bet expression in tumor. **(A)** The gene levels of TNF-α, IL-12, IFN-γ, and IL-6 were examined in tumor tissue from control and Astragaloside III treatment groups. **(B)** Gene level of T-bet but not GATA-3 was increased in tumor tissue of Astragaloside III-treated group. **(C)** Statistic data regarding the expression level of cytokine TNF-α, IL-12, IFN-γ, and IL-6 were examined in tumor tissue from control and Astragaloside III treatment groups. **(D)** Expressions of transcription factor T-bet and GATA-3 were detected in tumor tissue from control and Astragaloside III treatment groups. All data are presented as the means ± standard errors. * *P* < 0.05, ** *P* < 0.01, *** *P* < 0.001.

To further study the underlying mechanism of Astragaloside III on NK cells, we analyzed the gene expression of cytokines and transcription factors associated wih NK cell activation. As shown in **Figure 6A**, the gene expressions of IFN-γ, IL-12, and IL-6 but not TNF-α were increased in tumors of the Astragaloside III treatment group than in the control group. Moreover, Astragaloside III significantly upregulated the secretion of IFN-γ through upstream transcription factor T-bet but not GATA3 (**Figure 6B**, *P* < 0.01). The protein level of the abovementioned genes was further detected. The expressions of cytokine IFN-γ and IL-12 but not TNF-α and IL-6 were increased in tumors of the Astragaloside III treatment group than in the control group (**Figure 6C**). In addition, Astragaloside III significantly upregulated transcription factor expression of T-bet but not GATA3 (**Figure 6D**, *P* < 0.01). These data indicated that Astragaloside III could upregulate the expression of IFN-γ through the regulation of T-bet expression.

## Discussion

Colon cancer remains as major unmet medical need and a threat against human health. Several Chinese herbs have been used for treatment of cancer in ancient China, and Astragalosides belong to one class of these agents (Yan et al., 2017). Compounds from Astragaloside, such as Astragaloside II and IV, have been characterized with broad-spectrum antitumor activities (Cheng et al., 2014; Zhang et al., 2014; Dai et al., 2017; Hu et al., 2017; Jiang et al., 2017; Yan et al., 2017). However, the anti-tumor effect of Astragaloside III, an important constituent of Astragaloside, is largely unknown. In this study, we demonstrated that Astragaloside III significantly inhibited the growth of CT26 colorectal tumor through selectively increasing antitumor response of NK cell.

As Astragaloside is known to have an ability to regulate immune cells for tumor inhibition, NK cells and CT26 co-culture system were established to examine such functions of Astragaloside III. Indeed, Astragaloside III could significantly inhibit the proliferation of CT26 (**Figure 2A**) in cell coculture system. Follow-up studies in CT26-bearing mice model further demonstrated that Astragaloside III could hinder the tumor development *in vivo* by reducing tumor size and prolonging survival (**Figure 3**). In these and additional studies, the therapeutic effect was dose-dependent, and at a dose of 50 mg/kg body weight, no signs of toxicity (weight loss, elevated levels of ALT, and urine protein) were observed (data not shown). Unlike other Astragaloside components (Yan et al., 2017), Astragaloside III led to significantly increased numbers of NK cells in intratumoral regions (**Figure 4B**). As hepatic NK cells could be recruited into the tumor and play an important role in cancer therapies (Fang et al., 2017), the increased NK cells recruited by Astragaloside III from liver might mediate its anti-tumor effects.

It is known that NK receptors were important for the cytotoxic function of NK cells. Regulated by the negative regulatory factors in tumor microenvironment (Laouar et al., 2005), decreased levels of NKG2D expression impair the tumor-killing function of NK cells (Sun et al., 2012). To increase the anti-tumor response of NK cells, the expression of NKG2D needs to be elevated. In our study, Astragaloside III significantly increased the expression of NKG2D on NK cells *in vitro* (**Figure 1A**). CT26 and NK cell co-culture assay as well as CT26 animal model further proved that Astragaloside III effectively impeded tumor growth by increasing the expression of NKG2D (**Figure 2B** and **4C**). In addition to the observations in the tumor, Astragaloside III also significantly increased the expression of NKG2D on NK cells both in spleen and in lymph node (**Figure 5A**), indicating the broad-spectrum regulatory activity of Astragaloside III in general immunity. It is known that NKG2A is another classic NK receptor that negatively regulates cytotoxic function of NK cells (Angelini et al., 2011). Although Astragaloside III significantly upregulated the expression of NKG2D on NK cells, it showed no effect on NKG2A (**Figure S4B**). Therefore, the anti-tumor response of NK cells was enhanced by Astragaloside III through the increase of the expression of NKG2D.

Furthermore, NK cells can indirectly inhibit tumor growth and metastasis by secreting various effector molecules, such as IFN-γ (Pahl and Cerwenka, 2017). As cardinal IFN-γ producers, NK cell response is responsible for the pathogenesis of tumor. Increased IFN-γ production of NK cells has exhibited promising clinical benefits for patients with colon tumor (Cheng et al., 2013). Thus, the function of Astragaloside III on secretion of IFN-γ by NK cells was evaluated. We observed that IFN-γ production of NK cells was significantly increased after stimulation by Astragaloside III *in vitro* (**Figures 1B, D**), which reflects potential therapeutic mechanism of this compound. To further test this hypothesis, CT26 tumor cells were co-cultured with NK cells with or without Astragaloside III. Indeed, Astragaloside III significantly increased the production of IFN-γ by NK cells in co-culture system (**Figure 2C**). Moreover, Astragaloside III significantly increased the expression of Fas and Granzyme B but not Perforin on NK cells in cell coculture experiment. However, in animal model, expressions of Fas but not Granzyme B and Perforin were significantly increased on NK cells by Astragaloside III (**Figures 2D, E** and **4E**). CT26-bearing mouse model was also utilized to investigate the effect of Astragaloside III *in vivo*. Data from this study confirmed that Astragaloside III could significantly elevate the secretion of IFN-γ by NK cells both in tumor and in immune organs: spleen and lymph node (**Figures 4D** and **5D**). Besides IFN-γ, we also examined many other immune cytokines involved in the anti-tumor response of CT26, such as IL-12, from Th1 cells (Komai-Koma et al., 2016); IL-6 from antigen-presenting cells (Brahmakshatriya et al., 2017); and TNF-α from macrophages (Tessaro et al., 2017). Interestingly, Astragaloside III also strongly elevated the protein levels of IFN-γ, IL-12 but not TNF-a and IL-6 in tumor tissue, indicating the broad immune regulatory activities and restricted over inflammation in tumor. To probe the underlying mechanism of IFN-γ elevation, gene levels of transcription factors were analyzed. As we know, T-bet and GATA-3 were critical transcription factors for cytokine production balance (Kim et al., 2014), and their expression levels could regulate transcription levels of Th1 and Th2 response (Sun et al., 2018). In our study, we found that expression level of T-bet, but not GATA3, was significantly increased in Astragaloside III-treated group. These results together suggest that Astragaloside III regulates anti-tumor functions of NK cells through transcriptionally upregulating IFN-γ production by T-bet.

In this study, we, for the first time, elucidated the therapeutic effect of Astragaloside III in anti-tumor settings. Further studies are in progress to evaluate the bioabsorbance and pharmacokinetics of this compound. In view of the broad regulatory function of Astragaloside III mentioned above, other immune responses have also caught our interest, such as macrophage and Treg cell regulation. The effects of Astragaloside III in microenvironment of colon tumor need to be further studied. Given the potency and enriched source of Astragaloside III, application of this compound to tumor treatment might provide a lead candidate for novel therapeutic agent against colon cancer.

## Ethics Statement

This study was carried out in accordance with the recommendations of Animal Ethics Committee of the Tianjin International Joint Academy of Biotechnology and Medicine. The protocol was approved by the Tianjin International Joint Academy of Biotechnology and Medicine.

## Author Contributions

XMC and XC participated in the acquisition and analysis of data. JG, HY, and YD prepared the main figures and figure legend. YF and XH participated in editing the language and figures. XG and HW modified the article. XW and JC participated in the writing of the article and designed the study. All authors reviewed the article.

## Funding

This work was supported by a grant from National Natural Science Foundation of China (51573128, 31500723, and 31770974) for cell experiment. This work was also supported by a grant from Natural Science Foundation of Tianjin City (16JCQNJC13200,18JCZDJC34500) for mice experiment.

## Conflict of Interest Statement

The authors declare that the research was conducted in the absence of any commercial or financial relationships that could be construed as a potential conflict of interest.
