# Supplementary figures and images for: Astragaloside III Enhances Anti-Tumor Response of NK Cells by Elevating NKG2D and IFN-γ
Source: Front Pharmacol. 2019 Aug 13;10:898. doi: 10.3389/fphar.2019.00898 (PMC6701288; doi:10.3389/fphar.2019.00898)

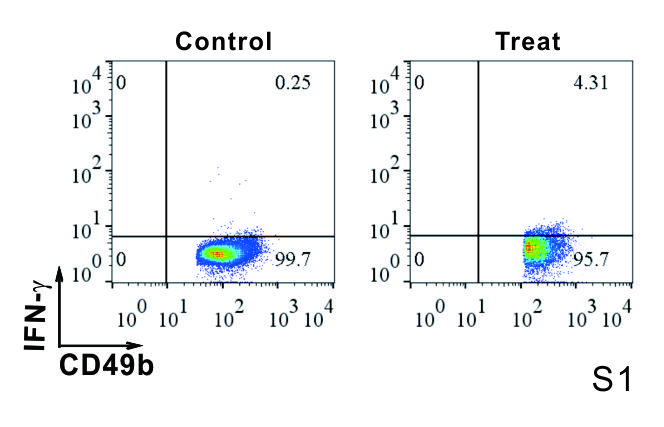

Supplement: Supplementary file 1 [file Image_1.jpeg]

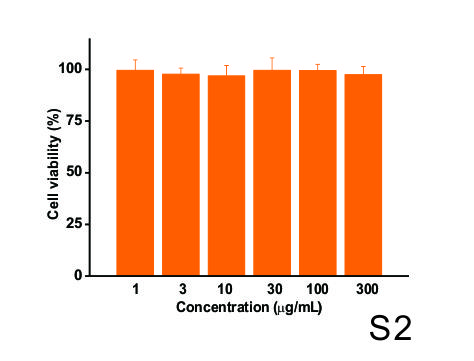

Supplement: Supplementary file 2 [file Image_2.jpeg]

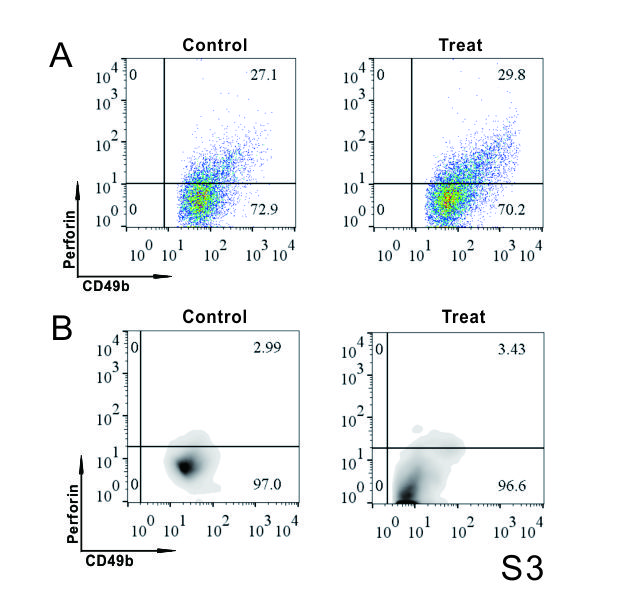

Supplement: Supplementary file 3 [file Image_3.jpeg]

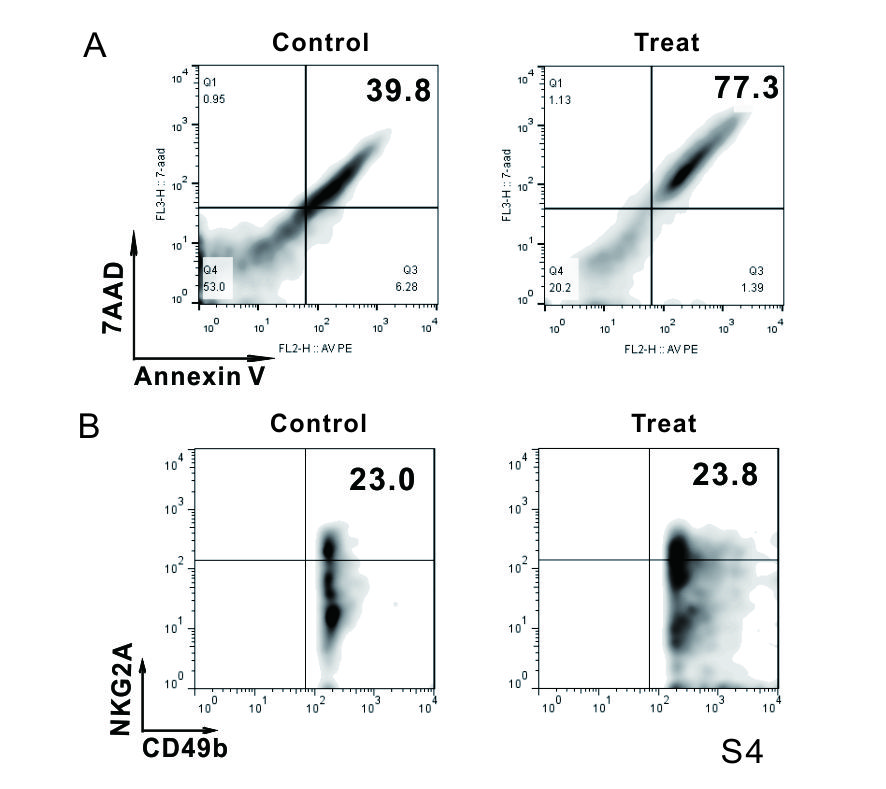

Supplement: Supplementary file 4 [file Image_4.jpeg]

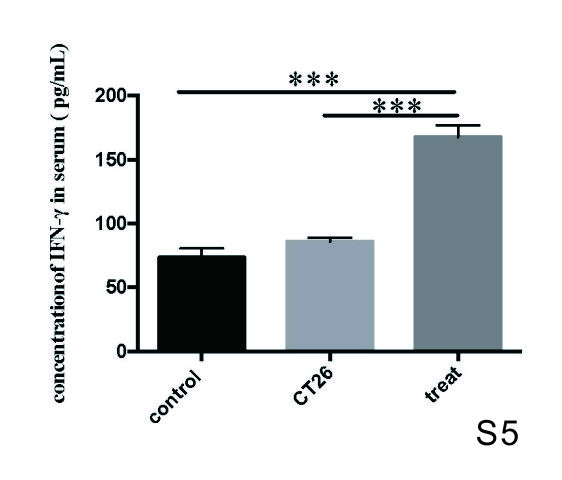

Supplement: Supplementary file 5 [file Image_5.jpeg]
