# Supplementary material for: Astragaloside III Enhances Anti-Tumor Response of NK Cells by Elevating NKG2D and IFN-γ
Source: Front Pharmacol. 2019 Aug 13;10:898. doi: 10.3389/fphar.2019.00898 (PMC6701288; doi:10.3389/fphar.2019.00898)
Supplement: Supplementary file 6 [file Table_1.docx]

**Supplementary Figure legends:**

Fig.S1 Secretion of IFN-γ by NK cells after treated with Astragaloside III.

Fig.S2 Viability of CT26 after treated with different dose of Astragaloside III.

Fig.S3 A. Expressions of Perforin on NK cells after co-cultured with CT26 cells were determined by flow cytometry. B. Expressions of Perforin on NK cells from CT26 tumor model were determined by flow cytometry.

Fig.S4 A. Apoptosis of CT26 cells after co-cultured with media of Astragaloside III activated NK cells. B. Expression of NKG2A on NK cells in control and Astragaloside III treatment group when cocultured with CT26.

Fig.S5 IFN-γ level in serum in animal model of control and Astragaloside III treatment group. All data are presented as the means ± standard errors. ***P<0.001
